# Placement of sutures for inside-out meniscal repair: both sutures through meniscal tissue reduces displacement on cyclical loading

**DOI:** 10.1186/s40634-021-00417-z

**Published:** 2021-10-21

**Authors:** Satoshi Yamakawa, Tatsuo Mae, Issei Ogasawara, Takehito Hirose, Shoji Konda, Ken Nakata

**Affiliations:** 1 Department of Sports Medical Biomechanics, Osaka University Graduate School of Medicine, 2-2, Yamada-oka, Suita, Osaka 565-0871 Japan; 2 Department of Health and Sport Sciences, Osaka University Graduate School of Medicine, Suita, Osaka Japan; 3 Department of Orthopaedic Surgery, Osaka University Graduate School of Medicine, Suita, Osaka Japan

**Keywords:** Meniscus, Repair, Inside-out technique, Longitudinal tear, Suture

## Abstract

**Purpose:**

The inside-out meniscal repair is widely performed to preserve the function of meniscus. In this technique, the outer suture is passed through the capsule as well as the outer meniscus, while the inner suture is inserted into the meniscus. The aim of this study was to biomechanically compare the suture stability between meniscus-meniscus and meniscus-capsule suture methods for the longitudinal meniscal tear with inside-out technique.

**Methods:**

Twenty-seven porcine knees were dissected to maintain the femur-medial capsule/meniscus-tibia complex, and the inner meniscus was cut off along the meniscus circumferential fiber with 3 mm width of the peripheral meniscus preserved. After one needle with a 2-0 polyester suture was inserted into the inner portion of the meniscus, the other needle was inserted through 1) the peripheral meniscus (Group A), 2) capsule just above the meniscus (Group B), and 3) capsule at 10 mm apart from the meniscus-capsule junction (Group C) in the inside-out manner. Then, the suture was manually tied on the capsule. The suture gap at the repair site during 300 times of cyclic loading and the ultimate failure load in the load-to-failure test were measured. The statistical significance of the data between two groups in each combination was considered by Bonferroni correction, following a one-way analysis of variance.

**Results:**

In the cyclic loading test, the suture gap was 0.68 ± 0.26 mm in Group A, 1.08 ± 0.36 mm in Group B, and 1.94 ± 0.57 mm in Group C with a significant difference. In the load-to-failure test, the ultimate failure load was 59.1 ± 13.6 N in Group A, 60.0 ± 7.9 N in Group B, and 57.4 ± 4.7 N in Group C, and there was no significant difference.

**Conclusion:**

The stitching region in the inside-out technique for longitudinal meniscal tear affected the stability of the tear site, and stitching the mid-substance region of the meniscus provides good stability in response to cyclic tensile loading. In addition, the stitching region did not affect the ultimate failure load.

**Clinical relevance:**

In the inside-out meniscal repair, the outer suture should be inserted into the remaining peripheral meniscus or the capsule near the meniscus.

## Introduction

The meniscus plays important roles such as shock absorption, joint stabilization, load transmission, and lubrication in the knee joint [2, 13, 19, 21, 26]. Due to its mechanical demand, the meniscus is frequently torn in athletic activities, and the frequency of meniscal injury is over 30% in total acute knee injury [22]. For treatment of the meniscal injury, the meniscectomy had been generally chosen [7, 14], however, the meniscectomy has found to result in the articular cartilage degeneration and osteoarthritis [16, 18]. Thus, the meniscal repair is proactively chosen for meniscal treatment to preserve the biomechanical function of meniscus [3–6, 10–12, 25].

Longitudinal meniscal tear in the red zone (blood supplied zone) is the best for meniscal repair, while the inside-out technique is commonly applied to meniscus repair [9].. As the longitudinal meniscal tears are observed not only at the mid-substance but also at the peripheral meniscus-synovium junction, one thread inserts into the meniscus inside of the tear and the other thread passes through the meniscus outside of the tear or the capsule, depending on the meniscus width outside of the tear. In case of meniscal repair including capsule, as the strength of the meniscus is quite different from the strength of capsule, the capsule can be damaged easily by suture material compared to the meniscus. However, the effect of the difference between stitching regions (meniscus-meniscus vs meniscus-capsule) on post-surgical stability is unclear, though the secure and strong suture method in meniscal repair is required to achieve satisfactory outcomes.

Previous studies evaluated the suture methods of the meniscus repair [1, 2, 20, 23]. Aık et al. compared the suture strength using bovine knees in meniscal repair among 5 suture techniques; horizontal mattress, vertical mattress, knot-end, vertical, and vertical loop, and described that the largest ultimate failure load occurred in the vertical mattress suture technique. However, as this report applied the tensile test to the meniscus without synovial capsule, ligaments and bones [1], the condition was different from the physiological state. Iuchi et al. firstly clarified the suture strength in meniscal repair for the physiological meniscus attached to the synovial capsules and bones [15]. Therefore, the aim of the present study was to biomechanically compare the suture stability between meniscus-meniscus and meniscus-capsule suture techniques for the longitudinal meniscal tear in the physiological condition. Our hypotheses were 1) the gap of suture site in the meniscus-capsular suture technique was larger than that in the meniscus-meniscus technique during the cyclic loading test and 2) the failure load did not differ between those techniques.

## Method

### Specimen preparation

**Fig. 1:**
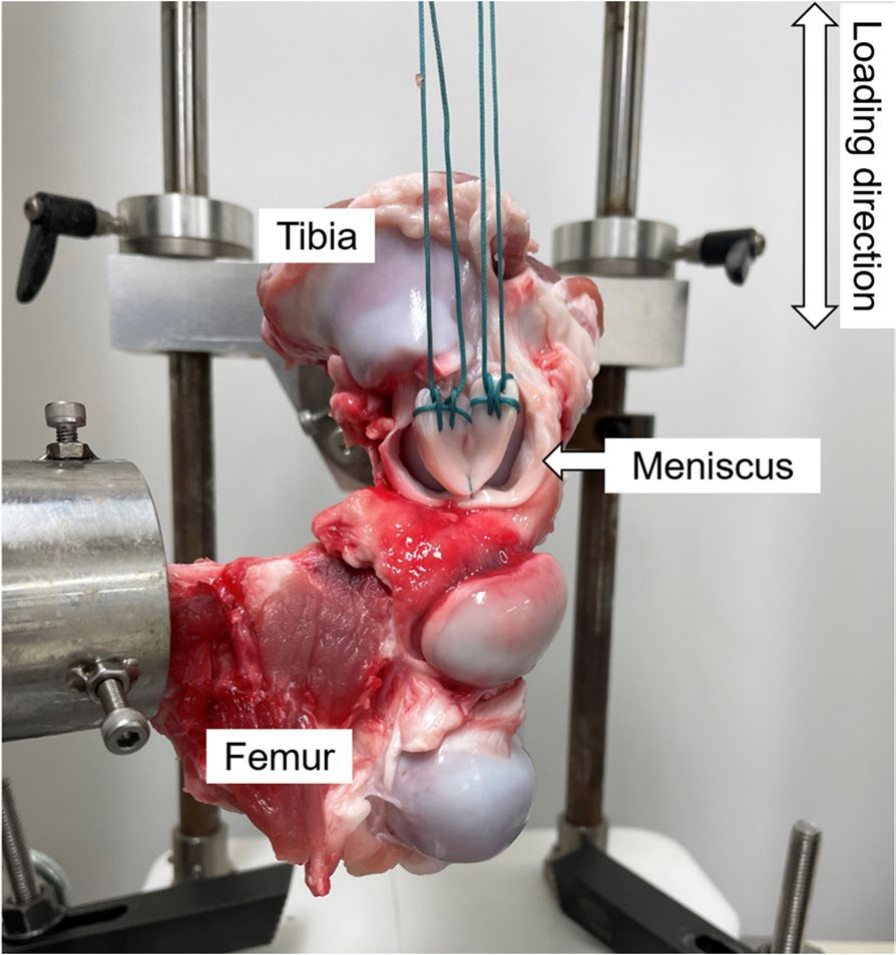
Specimen setup for the test. Femur and tibia were fixed to the bottom of the apparatus using custom-made jigs, while sutures on both ends of the meniscus were tied over the upper jig of the apparatus connected to the load cell

Twenty-seven porcine knee joints were used in the present study. The porcine were approximately 100 kg in weight with a mean age of 6 months. As all knee samples were obtained from the food industry and no animals were killed or sacrificed for this study, the study protocol was reviewed and determined not to require oversight by the institutional review board in our institutes. Specimens were stored in − 20 °C freezer and thawed with 4 °C temperature for 24 h prior to the test. All soft tissues around the knee joint were carefully dissected down while the medial meniscus and the medial joint capsule including medial collateral ligament were preserved. The inner portion of medial meniscus was sharply cut off along the meniscal longitudinal fiber with the scalpel while 3-mm width of the peripheral portion was preserved. Both ends of the excised meniscus were stitched by Krackow stitch using No.5 polyester sutures (Ethibond; EXCEL, ETHICON, Johnson & Johnson, USA) (Fig. 1).

### Meniscal repair

**Fig. 2:**
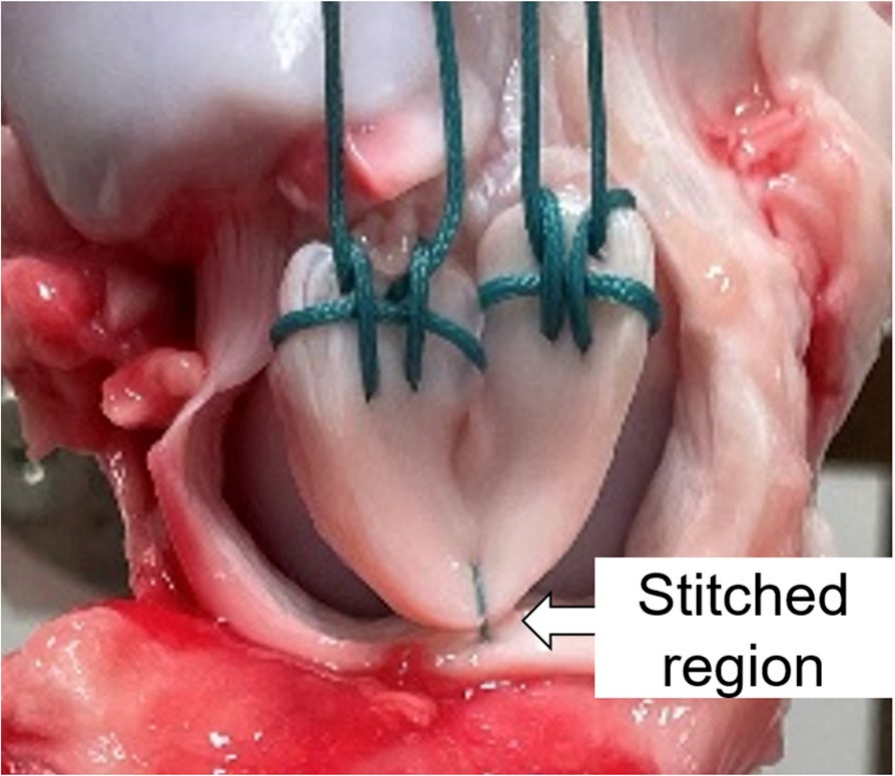
Suture placing for the longitudinal meniscus tear. A single stich was applied at the center of the excised meniscus on femoral side in the inside-out manner

**Fig. 3:**
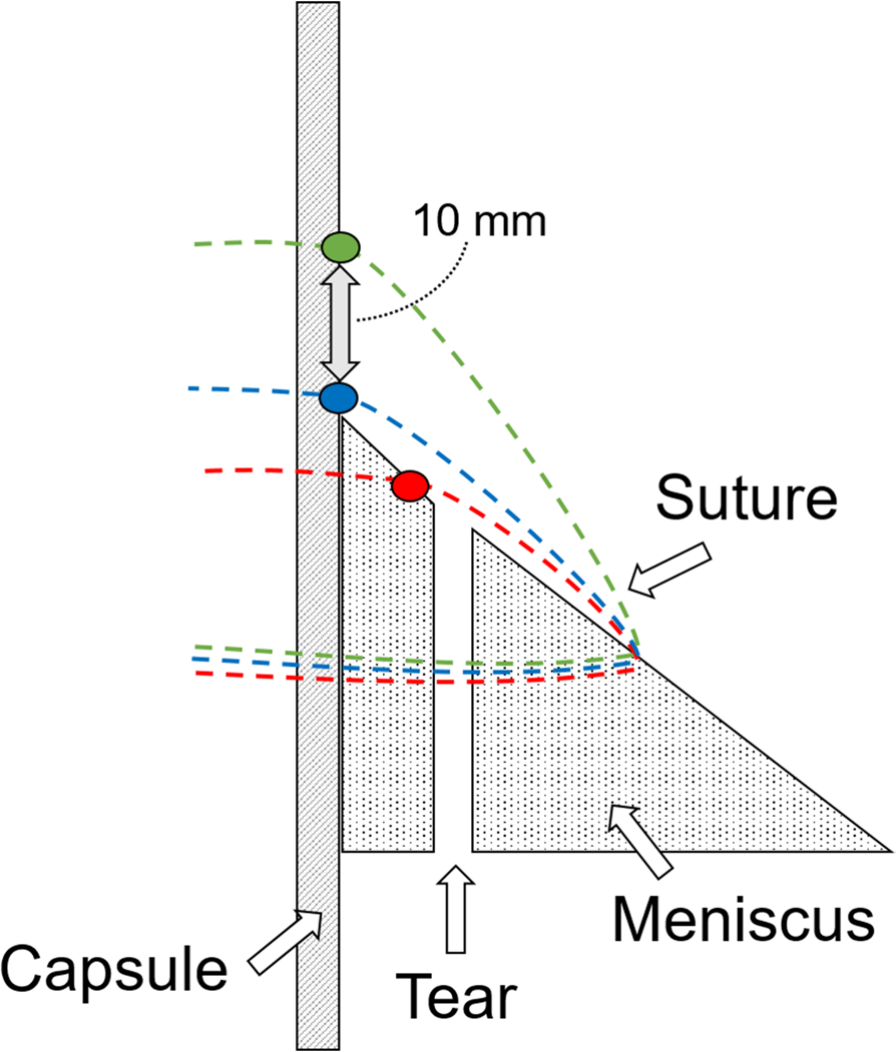
Stitching techniques. One needle was inserted into the meniscal body in all groups, while the other needle was inserted through 1) the peripheral meniscus (Group A: red dot), 2) capsule just above the meniscus (Group B: blue dot), and 3) capsule at 10 mm apart from the meniscus-capsule junction (Group C: green dot)

2-0 polyester sutures with two straight needles on both sides of a thread (Stryker Japan, Tokyo) were used for meniscal repair. Single stich was applied with this 2-0 polyester suture at the center of the excised meniscus on femoral side in the inside-out technique (Fig. 2). The specimens were divided into the following 3 groups (*n* = 9 to each group) based on the stitching technique. One needle was inserted at 3 mm apart from the outer site of the excised meniscus and was passed through the peripheral meniscus in all groups. The other needle was inserted through 1) the peripheral meniscus (Group A), 2) capsule just above the meniscus (Group B), and 3) capsule at 10 mm apart from the meniscus-capsule junction (Group C), respectively (Fig. 3). Then, the suture was manually tied on the capsule with four square knots at 30 degree of knee flexion. All knot tying were performed by one orthopaedic surgeon with more than 20-year experience (T.M).

### Biomechanical tests

Biomechanical tests were performed using material testing apparatus (AUTOGRAPH AG-IS, SHIMADZU, Kyoto, Japan). Femur and tibia were fixed to the bottom of the apparatus using custom-made jigs and No. 5 sutures on both ends of the excised meniscus were tied over the upper jig of the apparatus connected to the load cell. Then, the pre-conditioning was performed by applied 20 N of tensile load for 5 min. In the cyclic loading test, the cyclic load between 5 to 20 N with 20 mm/min velocity was applied 300 times followed the previous reports [8]. To assess the suture gap of the stitched site, suture insertion points were marked as a dot by surgical marker prior to the test, and both dots were recorded by a video camera (HDR-CX370V; SONY, Tokyo, Japan) during the test. Then, the motion of the dots during the test was tracked using a motion tracking system (Motion analyzer VW-9000, KEYENCE, Japan) based on the recorded movie. The gap between both dots was calculated before and after the test with a 5 N tensile force application state. After the cyclic loading test, the load-to-failure test was performed with 5 mm/min velocity referred to previous reports [15, 28], and recorded ultimate failure load and failure mode. All biomechanical tests were randomly performed among three groups.

### Statistical analysis

The study compared data among groups using a 1-way analysis of variance (ANOVA). Then, the statistical significance of the data between two groups in each combination was considered by Bonferroni correction, and it was set at *p* < 0.0167.

## Result

**Fig. 4:**
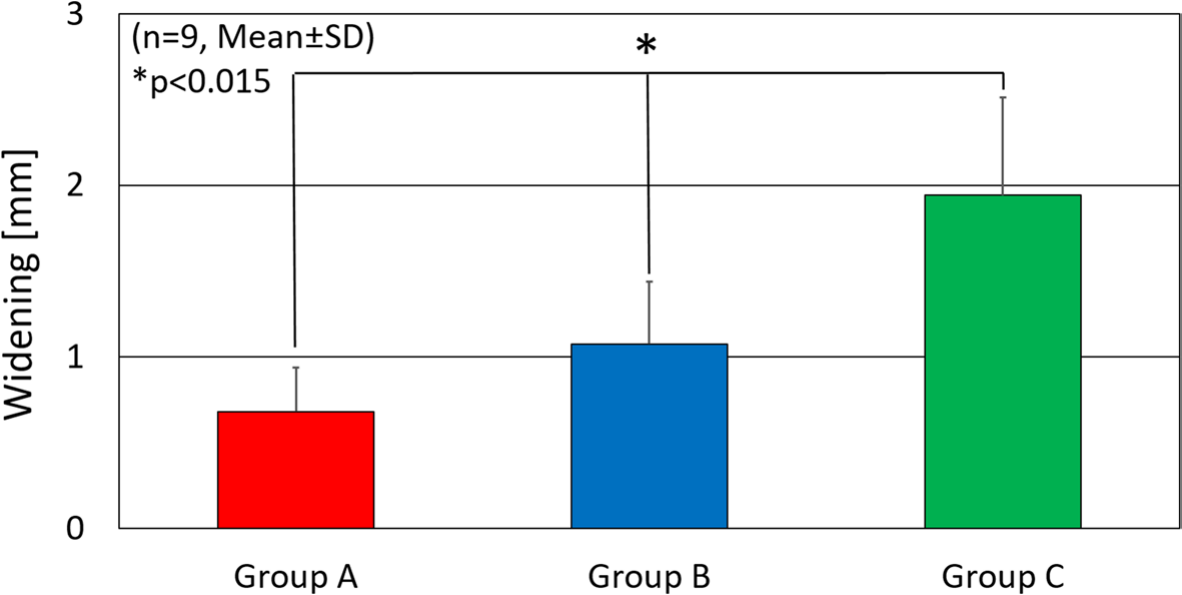
The gap at the suture site after 300 times of cyclic loading. There was a significant difference among three groups. * *p* < .0015

In the cyclic loading test, the suture gap was 0.68 ± 0.26 mm in Group A, 1.08 ± 0.36 mm in Group B, and 1.94 ± 0.57 mm in Group C respectively (Fig. 4). There was a significant difference between two groups in multiple comparisons. *P*-values in Group A vs B, Group A vs C and Group B vs C were 0.01, 0.001 and 0.002, respectively.

**Fig. 5:**
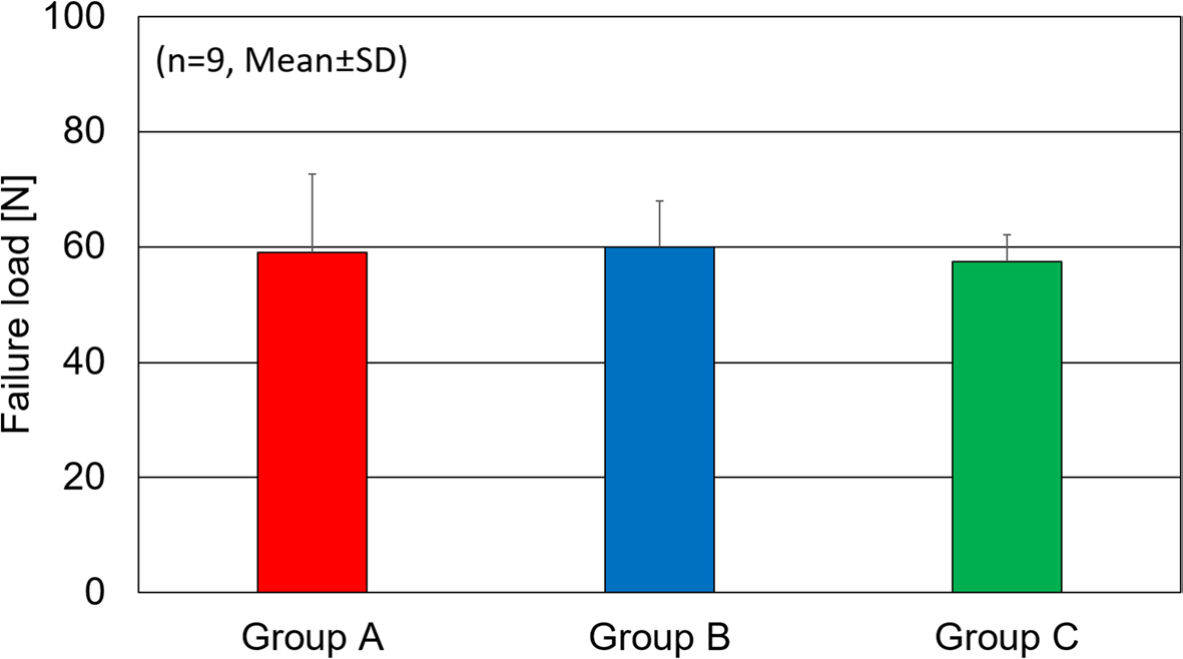
Ultimate failure load. There was no significant difference among three groups

In the load-to-failure test, the ultimate failure load was 59.1 ± 13.6 N in Group A, 60.0 ± 7.9 N in Group B, and 57.4 ± 4.7 N in Group C, respectively (Fig. 5). There was no significant difference between two groups in any combinations. *P*-values in Group A vs B, Group B vs C and Group A vs C were 0.8, 0.8 and 0.2, respectively. All specimens failed at the knot in ultimate failure load test.

## Discussion

The key finding of the present study was that the meniscus-capsule stitching method in the inside-out meniscal repair affects the suture gap of the repaired site during cyclic loading while there was no significant difference in the load-to-failure test.

The suture gap of the repaired site in Group B and C was significantly larger than that in Group A during cyclic loading. The cause of the suture gap widening was possible to be the damage of the tissue and the stitching structure. The meniscus is generally reinforced by collagen fiber [24], which is mainly oriented in a circular manner in the main region of the meniscus. Those fibers transmit the radial force to the circular manner and to the anterior and posterior horn ligament. In the present study, as the main fiber direction was perpendicular to the loading direction [24], the structure of the meniscus may have enough strength to the suture and its tensile loaded direction. On the other hand, although the joint capsule is also consisted by collagen fiber, its orientation is not homogeneous, and the strength to the loading direction was much smaller than the mid-substance of the meniscus [27]. Therefore, the capsule might be easier to be damaged by the suture material than the meniscus during the cyclic loading, and, in fact, a small tear occurred at the suture insertion point after the test.

There was no statistical difference in the ultimate failure load between each group, and all subjects in all groups showed knot failure. The previous reports also indicated that the failure mode in the load to failure test was mainly the knot failure [1, 2, 20, 23]. Then, the suture can be damaged in tying a knot the knot tying procedure for the meniscus repair may made a weak point in the stitching. Yokoi et al. indicated that the flat and wide shape suture material showed a higher ultimate failure load compared to conventional and hollow sutures [28]. Therefore, the suture material should be considered to keep the post-surgical stability of the stitched site. Moreover, in the clinical practice, multiple sutures are used for meniscal repair based on the size of the tear. Previously, Iuchi et al. reported that the multiple sutures generated much higher strength compared to the single suture in the meniscal repair [15]. Thus, meniscal repair with number of sutures can get enough strength in the surgical setup, though one suture was weaker than the meniscus tissue.

There were some limitations in this study. First, the porcine specimens were used for the test as a replacement for the human specimens. However, several previous studies reported that the porcine meniscus had comparable properties to those of human meniscus [8, 17], and the present testing setup was acceptable to determine the post-surgical stability of the stitched site in the meniscus repair. Second, the testing setup was only set in time zero, and the consequence of the stitched region in meniscus repair after surgery was not clarified. Third, the loading direction was not physiological, as range of motion exercise and weight bearing training are commonly performed in the post-operative rehabilitation program. However, the severe environment such as directly pulling the suture site could be important to detect the essential effect of suture technique. In future works, the physiological loading will apply to determine the magnitude of the suture gap. Finally, the point at which the needle came out from the capsule was not precisely assessed, although the insertion site of needle was evaluated. The needle direction after inserting to meniscus is also a key and should be controlled, as the needle direction can also affect the amount of capsule sutured behind the meniscus. However, the needle inserted to the mid-substance of the meniscus was direct-visually passed into the peripheral meniscus and was pushed to proximal. Thus, the effect of the amount of capsule behind the meniscus on suture gap could be minimized in the present study.

## Conclusion

The stitching region in the inside-out technique for longitudinal meniscal tear affected the stability of the tear site, and stitching the mid-substance region of the meniscus provides a good stability in response to cyclic tensile loading. In addition, the stitching region did not affect the ultimate failure load.
